# Global genetic fictions

**DOI:** 10.1136/medhum-2021-012236

**Published:** 2021-06-21

**Authors:** Clare Barker

This special issue has developed out of a research symposium on global genetic fictions, funded by Wellcome, which was held at the University of Leeds in April 2019. Involving historians, literary and cultural critics, it was a welcome opportunity to sample the wealth of humanities-based scholarship on genetic science, its cultural representations and its ethical implications. This is a field that is gathering momentum; the 2 years since that symposium, for instance, have seen the publication of three important literary critical books on genetics: Josie Gill’s *Biofictions: Race, Genetics and the Contemporary Novel;* Clare Hanson’s *Genetics and the Literary Imagination;* and Lara Choksey’s *Narrative in the Age of the Genome: Genetic Worlds*.^1^ This special issue seeks to consolidate this growing body of critical work on cultural representations of genetics and to further diversify its range of interests and applications. Collectively, we explore the circulation of ideas about genes, the genome and genetic science in cultural texts across a range of forms—from poetry to genre fiction, rap music to TED talks, popular science to historical fiction and postcolonial literature—and from diverse cultural locations.

Our focus on genetic *fictions* refers not only to the imaginative narratives found in literary texts, although many of the articles do draw on literature to make their arguments. Rather, following Gill’s conceptualisation of the ‘biofiction’ of race in genetic science, the ‘genetic fictions’ in this issue consist of the ideas about genetics (and related ideas about human identity, heredity, kinship, health and environments) that are ‘constituted through the complex entanglement of scientific and fictive forms’, with ‘fiction’ encompassing both literary texts and a more general sense of ‘the conjured up, the imaginary and the fictional’, the narratives about genes that are ‘formed in the political, social and cultural spheres’.^2^ This is important because, as biocultural critique has firmly established, cultural discourse *about* genetics does not just reflect, explain, interpret and popularise emerging scientific knowledge; it fundamentally contributes to the production of that ‘knowledge’ itself. A slightly earlier phase of scholarship conducted by historians of science and literary critics, which emerged alongside and in the wake of the Human Genome Project’s sequencing of the human genome (1990-2003), has demonstrated how the language and metaphors used to describe genes and the genome (to the general public and among scientific communities themselves) were integral to shaping the development of scientific disciplines and methods in genomic research. Linguistic, textual and informatic metaphors—’the “secret of life,” the code, the book, the alphabet, sentences, words, chapters, histories, the Rosetta stone, the Holy Grail, the recipe, the blueprint, the text, the map, the homunculus, software, and others’^3^—not only rendered complex biological processes comprehensible to lay audiences through journalism and popular science (and inevitably distorted them along the way), but also structured the conceptual frameworks and thus the research directions and priorities of molecular biologists, ‘both enabl[ing] and constrain[ing] the[ir] thoughts and actions’^4^.

More recently, Gill has shown how genetic fiction is ‘intimately and inextricably bound up in the formation of scientific fact, shaping and impacting upon the development, expression, transmission and ultimately the public understanding of the new science of race’, arguing persuasively that ‘fiction is part of truthmaking’.^5^ And as Everett Hamner puts it: ‘It is not that genetic fiction decides what scientists do in the lab on a day-to-day basis [...] rather [...] genetic fiction becomes a key background against which both scientists and the public imagine this work’s reper-cussions’.^6^ Some of the influential ‘genetic fictions’ considered and critiqued in this issue include, in Natalie Riley’s essay, the rise in the 1990s of a reductive brand of genetic determinism associated with neoDarwinist public figures such as Richard Dawkins; in Shital Pravinchandra’s article, the fiction of the purity and pending extinction of Indigenous DNA; and and how, as Lucy Burke demonstrates, life with disability is cast ‘as intrinsically diminshed and burdensome’ in bioethical discussion of genetic conditions such as Down’s syndrome.^7^ These are all pervasive and persistent narratives that shape public thought but also, crucially, contribute to scientific outcomes: the development of reproductive biotechnologies such as non-invasive prenatal testing is dependent on assumptions (fictions) about the quality of disabled lives, while priorities in population genetics are driven by the perceived need to capture ‘rare’ Indigenous genetic data before they are lost forever. As Hamner notes, the ‘mutated narratives’ that emerge in the translation of complex genomic science to its popular conceptualisations can ‘double back and colonize the research and applications that find private and public financing’.^8^ With this in mind, our essays pay particular attention to how the *form* of cultural texts operates to produce and to destabilise particular genetic fictions, from how, in Jerome de Groot’s article, intertextuality in contemporary poetry and the rhythm, rhyme and ‘spitting’ of rap lyrics creatively reform DNA test results to how, as Loredana Filip shows, the conventions of the TED talk as a genre of ‘self-improvement’ lend themselves to narratives of genetic enhancement or ‘self-fashioning’.

The entanglement and co-constitution between ‘fictional’ public discourses about genetics, scientific activity in the lab and research infrastructure point towards another central focus of this special issue. The human genome is, inevitably, a ‘global genome’ (to borrow Eugene Thacker’s term), since the infrastructure of the life sciences exists at a globalised level, bringing together state and corporate interests with international property law, global health priorities, and the transnational operations and markets of Big Pharma and the biotech industries.^9^ Both in material forms—cell samples moving between laboratories— and in the abstracted form of ‘data’ or information, the ‘exchange, circulation, and distribution of biological information and materials’ is one of the defining technologies of a globalised world.^10^ This produces distinct forms of economic valuation, or biocapital, meaning both that genomic science is inextricable from these forms of capitalist production and that life itself is continually ‘redefined through the contradictory processes of commodification’.^11^

In emphasising the *global* in the conceptualisation of this special issue, then, we aim to register the inequitable global distribution of biocapital and biotechnology, the unevenness of policies regarding benefit sharing, and the racial and cultural politics of genetic science. As Choksey points out, one preoccupation of ‘[n]arrative in the age of the genome’ is tracing how 21st century genomics positions human beings variously ‘as producer-consumers of data in the Global North or as peripheral and precarious manufacturers of technologies for interpreting these data in the Global South’.^12^ As several articles in this issue discuss, marketing for direct-to-consumer genetic ancestry testing has targeted Black and Indigenous communities whose ancestry or kinship ties may have been severed by the violence of slavery and settler colonialism.^13^ And major population genetics projects, which rely on the ‘distinctive’ genetic ‘data’ of Indigenous communities to trace human evolution, genetic diversity and histories of migration, have been censured for adopting ideological rationales and extractive methods of data collection that amount to contemporary forms of ‘biocolonialism’.^14^

Just as the uses and applications of genetic science are tied up with histories (and continuing presents) of racism, colonialism and exploitation, the global apprehension of genomics also differs according to diverse epistemologies and scientific practices. When Lily Kay pointed out in 2000 that the genetic imaginary according to which DNA is understood as ‘code’ and genes as vehicles for ‘information’ is ‘historically specific and culturally contingent’, ‘a manifestation of the emergence of the information age’—a particular facet of globalisation—she speculated that ‘there were (and probably could be) other ways of knowing’.^15^ As the articles in this issue demonstrate, there are indeed other ways of knowing—other frameworks for genetic knowledge, other conceptualisations of the human genome, identity, heredity, kinship, health and human-environment rela-tionality. Paul Hamann-Rose’s essay, for example, examines Amitav Ghosh’s representation of Indian ‘counterscience’ in The Calcutta Chromosome (1995), a fictional epistemological framework that destabilises the positivism of ‘Western’ knowledge systems and produces new genetic biotechnologies, and Frances Hemsley considers the epigenetic imaginary that emerges in the context of environmental racism in apartheid South Africa. To give another example, Maori (Ngāti Toa, Ngāti Raukawa and Te Āti Awa) novelist Patricia Grace describes genes as ‘the ancestors within us’ in Baby No-Eyes (1998), while Indigenous Science and Technology Studies scholar Kim TallBear (Sisseton-Wahpeton Oyate) writes that according to an ‘indigenous metaphysic’, ‘matter is lively’ or alive.^16^ To centralise Indigenous genetic epistemologies not only radically transforms narratives of ancestry, heredity and the inheritance of traits, but also holds significant implications for how genetic ‘samples’ should be stored, cared for and used.^17^

As Choksey argues emphatically in this issue, ‘Genomics is not a universal ontology, but a regional manifestation of a particular worldview that has become globalised and whose globalisation has been facilitated by violent processes of extraction’.^18^ But with the exception of recent studies such as Gill’s and Chok-sey’s monographs, most research on literary and cultural representations of genetics has so far focused on texts from the Global North that centralise EuroAmerican scientific epistemologies, with an understandable emphasis on the science fiction genre.^19^ In ‘Global Genetic Fictions’, we have collectively attempted to offer geographically diverse and culturally situated readings of genes, the genome and genetic science, drawing on cultural texts from varied locations, socioeconomic milieux and epistemological traditions. We are aware of Anne Whitehead and Angela Woods’ caution that globalising the medical humanities is not just about ‘expanding the canon of humanities texts that might be used in this context, to include postcolonial authors and/or works by indigenous writers’—although I believe this is important in a field in which a particular dominant scientific epistemology is often taken to be universal—but must necessarily also ‘contest or complicate a binary of “the West and the rest”, [and] think through in more complex terms the messy and uneven entanglement of subjects that globalisation inevitably entails’.^20^ In this special issue we hope to contribute to doing both, and Choksey’s pertinent question in the Introduction to *Narrative in the Age of the Genome* applies just as well to the concerns of many of the articles in this issue: ‘how does the messiness of what genomics does not account for — other histories, other forms of inheritance, other modes of being - interrupt and destabilize the seemingly implacable logic of cause and effect bound up in the idea of a molecular script?’^21^

In the articles to follow we see multiple examples of this messiness, which emerges in cultural texts that engage, incorporate and grapple with genetic discourse while also asserting the importance of what genomics does not account for, of alternative ways of seeing and knowing the world and being and thriving within it. The analysis in this issue therefore participates in the kind of politicised critical activity that Jenny Reardon has deemed characteristic of ‘the postgenomic condition’—the period since the sequencing of the human genome in which attention has turned from accumulating genetic knowledge to acts of interpretation and meaning-making. As Jay Clayton argues in his essay on ‘genome time’, tests as simple as a cheek swab can now render our past (our deep ancestry) and our future (susceptibility to disease) immediately knowable in the present moment, but the consequences of having this information at our fingertips, and the ethics of making such knowledge available to individuals and families (or indeed, medical practitioners, employers and the state), are still far from clear. Genetic fictions—both literary texts and pervasive cultural narratives—are important sites of encounter and entanglement, and the essays in this issue contribute to the ongoing project of postgenomic recalibration, contextualisation and reflection on what kinds of knowledge are necessary or desirable at a time when ‘people around the world are raising questions about how to know meaningful life on a data-rich but environmentally depleted, and interconnected-yet-fractured planet’.^22^

The articles in this issue are wide-ranging but share a number of key concerns. The first of these is how biotechnologies such as gene editing and prenatal diagnosis might ‘enhance’ or eradicate aspects of human diversity. Loredana Filip examines how the TED talk—one of the most ubiquitous and accessible global forms for the dissemination and popularisation of scientific knowledge—contributes to shaping global discourse about genetics. In the context of debates about genetic enhancement prompted by CRISPR-Cas9 and gene editing technologies, Filip argues that the TED talk can be situated within a tradition of ‘self-help and selfimprovement’, characterised by optimism, inspirational storytelling, and motivating narratives of success. TED talks on the possibilities of genetic science generate the sense of wonder that we often associate with science fiction and perpetuate narratives of visionary scientific genius. But Filip finds that several influential talks bypass concerns about privacy and ethics and espouse ableist assumptions about the desirability of genetic enhancement. Ultimately, the culture of self-fashioning that forms the TED genre also shapes the portrayal of gene editing itself as an exciting new mode of self-improvement. As Filip demonstrates, ‘seemingly harmless rhetorical choices’ in popular cultural forms ‘may all contribute to the rise of human enhancement ideals’.^23^

Exploring another angle on the eugenic potential of genetic knowledge, Lucy Burke makes a powerful argument about how ‘the history of genomic discourse is entangled with biopolitical and economic considerations that subject the value of some lives to the instrumentalist logic of the cost/benefit analysis’.^24^ Burke unpacks debates within health economics and reproductive bioethics about prenatal diagnosis and selective abortion in relation to genetic conditions including Down’s syndrome. All too frequently, she demonstrates, these discussions pivot on unevidenced and ableist assumptions about burdensome disabled children and suffering mothers. Burke then turns to Icelandic author Yrsa Sigurdardóttir’s crime novel Someone To Watch Over Me (2013). Set in the aftermath of the global financial crash of 2008 and concerned with questions of state power, truth and justice, this text offers an alternative view on human value. When the resolution of the crime narrative is achieved through the Foucauldian ‘subjugated knowledges’ of characters with learning disabilities, Burke argues that the novel profoundly challenges neo-utilitarian approaches to prenatal diagnosis. Framing her arguments with feminist disability studies perspectives, Burke exposes and problematises the economic logic surrounding reproductive technologies that ‘render some groups of people with genetic conditions existentially vulnerable’.^25^

Other essays are concerned with the histories of genetic science and the temporalities of genetic knowledge. Jay Clayton elaborates on his previously articulated concept of ‘genome time’, the notion that genomics has produced a new, distinctive temporality, characterised by both simultaneity and linearity, presentness and the ongoingness of history, which promises ‘that all times will become discoverable in the present’.^26^ These temporal formations are not simply theoretical models but impact directly on public policy and consumer behaviour, as demonstrated by the thriving markets for both direct-to-consumer genetic ancestry research and genetic health risk testing. Clayton considers genome time in relation to the different temporal implications of nanotechnology and climate science, and through a queer reading of a classic science fiction short story, Samuel R Delaney’s Time Considered as a Helix of SemiPrecious Stones (1969), he argues that genome time can be aligned with forms of queer temporality, constituted not necessarily by deterministic logic or foreclosed futures but by notions of potentiality and possibility.

Jerome de Groot is interested in the contemporaneity and historicity of genomic artefacts—both material DNA samples and the creative texts that represent and explore the postgenomic condition. He analyses the postgenomic imaginaries on display in the 21st century art of Marc Quinn, poetry of Zaffar Kunial, Michael Symmons Roberts and Hannah Sullivan, and the rap lyrics of Kendrick Lamar and Residente. This diverse group of creative practitioners variously reflects on past modes of knowing the human— ‘the resonance of the then in the now’— through intertextual ‘updates’, formal innovations, translations and adaptations of their literary and musical inheritances.^27^ In poetry and lyrics about DNA, they also testify to the historical specificities of their own, postgenomic, moment, forging new ways of engaging with the self, the other, ancestry, kinship, race, ethnicity and the materiality of the human body in relation to their new genetic knowledge. Like Clayton, de Groot identifies opportunities for innovation, creativity and critique within these postgenomic imaginaries: the writers he considers ‘are not inflected by their genetics but rather make the possibilities work for them’.^28^

Natalie Riley’s article is also centrally concerned with the relationship between genetic science and history, focusing on the 1990s—the decade of the Human Genome Project—and the neo-Darwinian accounts of human heredity and behaviour popularised by public intellectuals such as Richard Dawkins and Steven Pinker. Riley argues that in contrast with the genetic determinism of these narratives, three British novels—A.S. Byatt’s *Babel Tower* (1996), Zadie Smith’s *White Teeth* (2000) and Jenny Diski’s *Monkey’s Uncle* (1994)—mobilise the formal features of historical fiction, including anachronism, repetition and asynchronicity, as ‘a means of critiquing and destabilising the explanatory promise of human genomics’.^29^ Respectively engaging with linguistic nativism (the idea that a capacity for language is hard-wired in the human brain), the continuing presence of scientific racism within contemporary bioengineering, and reductive genomic explanations for depression, these texts share a sense of genomic scepticism. Riley demonstrates how, by drawing attention to the importance of context, translation and interpretation in making meaning out of ‘data’, these 1990s texts ‘highlight how the logics and cultural capital of genetics has always been, and must continue to be, disrupted, refused, and undermined’.^30^

Many of the articles in this issue engage with postgenomic questions regarding ancestry, race and inheritance, questions that have also long been central to the concerns of postcolonial literature. Paul Hamann-Rose explores narratives of genetic kinship that emerge in Amitav Ghosh’s *The Calcutta Chromosome* (1995) and Zadie Smith’s *White Teeth* (2000). Since genetic science (and ancestry testing in particular) has produced new ways to trace migration patterns around the globe, the concept of genetic kinship has special relevance to the articulation of postcolonial and diasporic narratives of home, family, relatedness and belonging in a globalised world. In *White Teeth*, which navigates between London, Bangladesh and Jamaica, genetic discourse is deployed by characters to ‘fortify a cultural and national continuity across the gulf of globalisation and diaspora’—not always successfully—while in *The Calcutta Chromosome*, Indian counterscience produces new genetic biotechnologies that allow the personalities of individuals from different ethnic and national backgrounds to be fused within a single ‘geneticised diasporic body’.^31^ Hamann-Rose argues that discourses of genetic kinship offer to postcolonial writers a new facet to the production of cultural identity in diaspora, and in turn postcolonial literatures demonstrate how kinship cannot ultimately be reducible to biological relations.

Kinship is also central to Shital Pravin-chandra’s analysis of Indigenous counter-genetic fictions. She explores an emerging trope in fiction and film, in which Indigenous bodies are immune to ‘an array of maladies plaguing all other humans’, and argues that ‘the idea of the biomed-ically distinctive Indigenous Person as a preyed-upon asset’ responds directly to the extractive logic of population genetics projects.^32^ Metis author Cherie Dimaline’s young adult novel *The Marrow Thieves* (2017) is set in a near future in which the Earth has been ravaged by climate change and all non-Indigenous humans have mysteriously lost the ability to dream. In this context, indigeneity has acquired a particular form of desirable biovalue since the ‘greater good’ of the human race now depends on the forcible extraction of Indigenous bone marrow. Pravinchandra shows how Dimaline employs genetic framings of identity and relationality alongside ‘more than biological’ Indigenous articulations of self-recognition, kinship and heredity in order to expose the limitations of genetic discourse. Importantly, she argues, the novel’s affirmation of queer kinship confirms ‘the complexities of Indigenous kin-making’ and reorients ‘tradition’ to ensure community survivance while resisting the heteroreproductive logic of biological heredity.^33^

The rapidly diversifying field of epigenetics, ‘the study of mechanisms that regulate gene expression in response to environmental signals’, has profoundly complicated ‘the gene-centrism and genetic reductionism of the genomic age’ and as such constitutes ‘an archetypal postgenomic science’.^34^ We are much more interdependent with our environments (from cellular to social levels) than previous science ever realised. But the implications of epigenetic research for social, environmental and racial justice movements are complex and messy, with the potential both to challenge and to entrench difference and stigmatisation. Frances Hemsley examines the potential for epigenetics to ‘allow us to better conceptualise the biopsycho-socially constitutive nature of racist environments’ while acknowledging the risk that it simultaneously holds the potential to ‘pathologis[e] particular social or ethnic groups as biologically damaged’.^35^ The racist environments in question in Hemsley’s article are the racially segregated slum areas of Cape Town in apartheid South Africa, spaces of poverty that both materialise and perpetuate the ideology of apartheid. Hemsley analyses Bessie Head’s *The Cardinals*, a novella written in the early 1960s in Cape Town’s District Six. While this text predates scientific research into epigenetics, Head generates an ‘epigenetic imaginary’ that interrogates the intersections of race, heredity and environment, complicating these entanglements through creative modes of self-invention and an incest narrative that emphasises the complexity of development. Hemsley concludes with appropriate caution that epigenetics ‘may still prove conceptually useful to postcolonial politics, environmental justice and antiracism’, but to do so it must operate in tandem with a critique of ‘material inequality’.^36^

Lara Choksey examines what it is possible to know about environmental ‘influence’ in the context of epigenetic research. While epigenetics may trouble models of genetic determinism that seek to produce categories of ‘race’, and while it may hold reparative potential in contexts of health inequalities, Choksey evidences the ways in which epigenetic studies may also ‘participate in processes of environmental racialisation’: ‘The logic moves from the racialisation of defect to the racialisation of defective influence’, and environments become racialised ‘through imaginaries of bad influence’.^37^ She then discusses Jay Bernard’s ‘Chemical’ (2019), a poetic testimony to, and elegy for, the death of residents (many of whom were not British citizens) in the 2017 Grenfell Tower fire in West London. Reading ‘Chemical’ alongside the state’s problematic enquiry into soil contamination around Grenfell, Choksey demonstrates how Grenfell’s surviving residents have been subjected to state-endorsed environmental racialisation. The ghostly presences in Bernard’s poem, in contrast, work against such fixing and ‘stay [...] with the dead’ in ways that suggest ‘the messiness of entanglements between the living and the dead, as well as their endurance’, and that emphasise ‘influence, reparation and responsibility’.^38^

The articles collected in this special issue testify to the vibrancy and vision of creative genetic imaginaries. Genetic fictions show how epistemological, political and bioeth-ical debates play out in the context of real and imagined lives and relations, and allow different ways of knowing to exist alongside one another. With this special issue we hope to contribute to the growing momentum in a rich area of the critical medical humanities, in particular by emphasising the diversity of epistemological frameworks for apprehending the applications and implications of genetic science. The relationship between genetic fictions and emerging bioscientific knowledge may be one of commentary, reflection, influence, critique, affirmation, inspiration, amplification and/or distortion, but there is no doubt that cultural texts and their critical analysis have much to offer in the unfolding context of postgenomic meaning-making.

## Footnotes

1
Josie Gill (2020), *Biofictions: Race, Genetics and the Contemporary Novel* (London: Bloomsbury); Clare Hanson (2020), *Genetics and the Literary Imagination* (Oxford: Oxford University Press); and Lara Choksey (2021), *Narrative in the Age of the Genome: Genetic Worlds* (London: Bloomsbury).

2 Gill, *Biofictions*, 5, 4.

3
Judith Roof (2007), *The Poetics of DNA* (Minneapolis and London: University of Minnesota Press), 7. See also Evelyn Fox Keller (1995), *Refiguring Life: Metaphors*

*of Twentieth-Century Biology* (New York: Columbia University Press); and Evelyn Fox Keller (2002), *Making Sense of Life: Explaining Biological Development with Models, Metaphors and Machines* (Harvard, MA: Harvard University Press).

4
Lily E Kay (2000), *Who Wrote the Book of Life? A History of the Genetic Code* (Stanford, CA: Stanford University Press), xviii. Kay goes on: ‘once molecular biologists adopted the scriptural representations of the genetic code, once they committed themselves, consciously or not, to the information discourse and to the attendant analogies of genomic writing and reading, these representations became constitutive of the decoders’ reasoning; their work was shaped by the new biosemiotics of communication’ (xviii). Hanson cautions that ‘Metaphors are intrinsic to scientific thought, enabling novel conceptualizations of the material world, but become problematic when they are used in the crossover genre of popular science’. Hanson, *Genetics and the Literary Imagination*, 10.

5 Gill, *Biofictions*, 4, 19.

6
Everett Hamner (2017), *Editing the Soul: Science and Fiction in the Genome Age* (Pennsylvania: Pennsylvania State University Press), 3.

7
Lucy Burke (2021), “Hostile Environments? Down’s Syndrome and Genetic Screening in Contemporary Culture,” *Medical Humanities* 47, no. 2: 193-200.

8 Hamner, *Editing the Soul*, 4.

9 See Eugene Thacker (2005), *The Global Genome: Biotechnology, Politics, and Culture* (Cambridge, MA and London: MIT Press).

10 Thacker, *The Global Genome*, 6.

11
Kaushik Sunder Rajan (2006), *Biocapital: The Constitution of PostgenomicLife* (Durham, MA and London: Duke University Press), 47. See also Melinda Cooper (2008), *Life as Surplus: Biotechnology and Capitalism in the Neoliberal Era* (Seattle: University of Washington Press); and Nikolas Rose (2007), *The Politics of Life Itself: Biomedicine, Power, and Subjectivity in the Twenty-First Century* (Princeton, NJ and London: Princeton University Press).

12 Choksey, *Narrative in the Age of the Genome*, 5.

13 See Kim TallBear (2013), *Native American DNA: Tribal Belonging and the False Promise of Genetic Science* (Minneapolis: University of Minnesota Press); and Alondra Nelson (2016), *The Social Life of DNA: Race, Reparations, and Reconciliation After the Genome* (Boston, MA: Beacon Press).

14 On biocolonialism, see Indigenous Peoples Council on Biocolonialism (2000), “Indigenous Peoples, Genes and Genetics: What Indigenous Peoples Should Know About Biocolonialism. A Primer and Resource Guide,” http://www.ipcb.org/publications/primers/htmls/ipgg.html; and Laurelyn Whitt (2009), *Science, Colonialism, and Indigenous Peoples: The Cultural Politics of Law and Knowledge* (Cambridge: Cambridge University Press). Indigenous geneticists are producing new research paradigms for ethically engaged genomic research; see, for example, Katrina G Claw et al. (2018), “A Framework for Enhancing Ethical Genomic Research with Indigenous Communities,” *Nature Communications* 9, no. 1: 2957, https://doi.org/10.1038/s41467-018-05188-3.

15 Kay, *Who Wrote the Book of Life?*, 2.

16
Patricia Grace (1998), *Baby No-Eyes* (Honolulu: University of Hawai’i Press), 280; Kim TallBear (2017), “Beyond the Life/Not-Life Binary: A Feminist-Indigenous Reading of Cryopreservation, Interspecies Thinking, and the New Materialisms,” in *Cryopolitics: Frozen Life in* *a Melting World*, eds. Joanna Radin and Emma Kowal (Cambridge, MA and London: MIT Press), 179–202 (199).

17 TallBear, “Beyond the Life/Not-Life Binary.” On the storage of Indigenous genetic biomatter, see also Joanna Radin (2017), *Life on Ice: A History of New Uses for Cold Blood* (Chicago and London: University of Chicago Press).

18
Lara Choksey (2021), “Environmental Racialisation and Poetics of Influence in the Postgenomic Era: Fire, Soil, Spirit,” *Medical Humanities* 47, no. 2: 145–55.

19 In a survey of the literature of genetics, Roof notes that the ‘majority of genetic fictions’ are science fictional and ‘focus on the possibilities of cloning, genetic engineering, and mutations’. Judith Roof (2011), “Genetics,” in *The Routledge Companion to Literature and Science*, eds. Bruce Clarke and Manuela Rossini (London and New York: Routledge), 124–34 (128). Jackie Stacey (2010), *The Cinematic Life of the Gene* (Durham, NC and London: Duke University Press), analyses a series of feature films that broadly sit within the science fiction genre.

20
Anne Whitehead and Angela Woods (2016), “Introduction,” in *The Edinburgh Companion to the Critical Medical Humanities*, eds. Anne Whitehead and Angela Woods (Edinburgh: Edinburgh University Press), 1–31 (21). Writing 10 years ago, Claire Hooker and Estelle Noonan identified how ‘medical humanities as a field has often been strongly, although not wholly, reflective of the traditions of Western (Anglo-American and European) culture, particularly what used to be referred to as “high” culture’. Claire Hooker and Estelle Noonan (2011), “Medical Humanities as Expressive of Western Culture,” *Medical Humanities* 37, no. 2: 79–84 (79). Our coverage of popular cultural forms as well as literary fiction is an attempt to help address both aspects of this critique.

21 Choksey, *Narrative in the Age of the Genome*, 5.

22
Jenny Reardon (2017), *The Postgenomic Condition: Ethics, Justice and Knowledge after the Genome* (Chicago and London: University of Chicago Press), 4. It is important to note, as Choksey does here, that ‘A decolonial approach would be to consider the postgenomic moment to be as regional to Eurocentric knowledge systems as the Human Genome Project was’. Choksey, “Environmental Racialisation and Poetics of Influence in the Postgenomic Era”. Its revisions and recalibrations, such as epigenetic understandings of the imbrication of organism and environment, for example, may be far from revelatory to many commentators in the Global South in particular, for whom such current science is providing alternative forms of evidence, and a different epistemological framework, for knowledge that has long existed. For an elaboration of this argument regarding Indigenous knowledge and epigenetics, see Emma Kowal and Megan Warin (2018), “Anthropology, Indigeneity, and the Epigenome,” *American Anthropologist* 120, no. 4: 822–5.

23
Loredana Filip (2021), “Genetic Enhancement, TED Talks, and the Sense of Wonder,” *Medical Humanities* 47, no. 2: 210–8.

24 Burke, “Hostile Environments,” 193–200.

25 Burke, 193–200.

26
Jay Clayton (2021), “Time Considered as a Helix of Infinite Possibilities,” *Medical Humanities* 47, no. 2: pages. See also Jay Clayton (2013), “Genome Time: Post-Darwinism Then and Now,” *Critical Quarterly* 55, no. 1: 57–74.

27
Jerome de Groot (2021), “History, Pastness, and the Postgenomic Imaginary,” *Medical Humanities* 47, no. 2: 177–84.

28 de Groot, “History, Pastness, and the Postgenomic Imaginary,” 177–84.

29
Natalie Riley (2021), “Out of Date: Genetics, History and the British Novel of the 1990s,” *Medical Humanities* 47, no. 2: 201–9.

30 Riley, “Out of Date,” 201–9.

31
Paul Hamann-Rose (2021), “New Poetics of Postcolonial Relations: Global Genetic Kinship in Zadie Smith’s *White Teeth* and Amitav Ghosh’s *The Calcutta Chromosome,” Medical Humanities* 47, no. 2: 167–76.

32
Shital Pravinchandra (2021), “More Than Biological: Cherie Dimaline’s *The Marrow Thieves* as Indigenous Counter-Genetic Fiction,” *Medical Humanities* 47, no. 2: 135–44.

33 Pravinchandra, “More Than Biological,” 135–44. As Pravinchandra explains, ‘survivance’ is Anishinaabe critic Gerald Vizenor’s term for active Indigenous practices of resistance to settler dominance. See Gerald Vizenor (2008), “Aesthetics of Survivance,” in *Survivance: Narratives of Native Presence*, ed. Gerald Vizenor (Lincoln: University of Nebraska Press), 1–23.

34
Hallam Stevens and Sarah S Richardson (2015), “Beyond the Genome,” in *Postgenomics: Perspectives on Biology After the Genome*, eds. Sarah S. Richardson and Hallam Stevens (Durham, NC and London: Duke University Press), 1–8 (4, 3, 4).

35
Frances Hemsley (2021), “Reading Heredity in Racist Environments: Epigenetic Imaginaries in Bessie Head’s *The Cardinals,” Medical Humanities* 47, no. 2: 156–66. On such debates about epigenetics and racism, see also Gill, *Biofictions*, chapter 5.

36 Hemsley, “Reading Heredity in Racist Environments,” 156–66.

37 Choksey, “Environmental Racialisation and Poetics of Influence in the Postgenomic Era,” 145–55.

38 Choksey, 145–55.
